# Information needs and information seeking behaviour of people with dementia and their non-professional caregivers: a scoping review

**DOI:** 10.1186/s12877-020-1454-y

**Published:** 2020-02-14

**Authors:** Aijia Soong, Shu Ting Au, Bhone Myint Kyaw, Yin Leng Theng, Lorainne Tudor Car

**Affiliations:** 1 Family Medicine and Primary Care, Lee Kong Chian School of Medicine, Nanyang Technological University Singapore, 11 Mandalay Road, Level 18, Clinical Science Building, Singapore, 308232 Singapore; 2 Lee Kong Chian School of Medicine, Nanyang Technological University Singapore, Singapore, Singapore; 3 Centre for Healthy and Sustainable Cities, Nanyang Technological University Singapore, Singapore, Singapore; 4 Department of Primary Care and Public Health, School of Public Health, Imperial College London, London, UK

**Keywords:** Information needs, Dementia, Informal caregivers, Information seeking behaviour, Review

## Abstract

**Background:**

People with dementia often require full-time caregivers especially in the later stages of their condition. People with dementia and caregivers’ access to reliable information on dementia is essential as it may have an important impact on patient care and quality of life. This study aims to provide an overview of the information needs and information seeking behaviour of people with dementia and their non-professional caregivers.

**Methods:**

We conducted a scoping review of the literature and searched four electronic databases for eligible studies published up to August 2018. Two reviewers independently screened studies and extracted data. Information needs were classified according to emerging themes in the literature, and information seeking behaviour was categorized using Wilson’s model of information behaviour.

**Results:**

Twenty studies with a total of 4140 participants, were included in this review. Reported information needs focused on: (i) disease; (ii) patient care provision; (iii) healthcare services; and (iv) caregiver self-care. The most commonly reported information need was on healthcare service-related information. Characteristics found to influence information needs were the severity of dementia as well as patient and caregiver status. People with dementia and non-professional caregivers mainly displayed active searching, information seeking behaviour and preferred using electronic sources to obtain health information.

**Conclusion:**

Current dementia information sources available in English are extensive in the information they offer, but more emphasis needs to be placed on healthcare service-related information. All studies originated from high income countries and focused on information needs of non-professional caregivers only. The only variables found to be associated to information needs were severity of dementia condition as well as patient/caregiver status. The information needs identified in this review can be used to inform development and design of future dementia resources for people with dementia and their non-professional caregivers.

## Background

The world’s population is steadily ageing, with a corresponding increase in the prevalence of dementia and its disease burden [1, 2]. There are approximately 50 million people living with dementia worldwide, with this estimate set to rise to 152 million by 2050 [3]. Dementia prevalence varies by geographic regions, with East Asia being the region with the largest number of people living with dementia, followed by Western Europe, South Asia and North America [1]. The cost of dementia care is limited not just to financial burden, estimated at US$818 billion globally in 2015, but also extends to the caregiver’s physical, psychological and social strain [4, 5]. As dementia progresses, people with dementia lose the ability to care for themselves and independently perform activities of daily living (ADLs) such as bathing, doing household chores, and cooking. Many persons with dementia would thus require full-time caregivers in the advanced stages of the disease, most of whom are their family members providing informal care [6].

Existing literature focuses on dementia caregivers and healthcare service needs rather than information needs [7–10]. Education of both patient and caregiver and provision of information is crucial in the long-term management of dementia. Most persons with dementia will eventually require a caregiver in the latter stages of the condition. Majority of these caregivers are informal caregivers without prior knowledge or training in dementia care. Providing access to relevant and reliable information to these caregivers is essential to ensure continued high-quality care for the person with dementia. The most recent studies investigating information needs for persons with dementia and their caregivers reveal that these groups do not receive sufficient information, emphasizing a need to identify information that these groups require [11–13]. There is some evidence pointing to the need for information on management of the behavioural and psychological symptoms of dementia as well as information on legal and financial issues along the course of disease progression [14, 15].

Existing reviews mostly focus on caregivers, their needs in general, or interventions to address caregiver needs, or have been undertaken more than 10 years ago [8, 10, 16]. In this scoping review, we aim to provide a broad scope of the current evidence by including perspectives of both people with dementia and non-professional caregivers, without any restrictions on publication date, to summarise and identify the information needs and information seeking behaviour of people with dementia and their non-professional caregivers. We were also interested in identifying the types of available evidence, key concepts, factors associated with information needs and information seeking behaviour as well as gaps in the existing literature. Correspondingly, we used scoping review methodology in order to fulfil the aim of providing a broad overview of all information needs and information seeking behaviour, and to determine any unmet information needs and guide development of future interventions.

## Methods

### Study selection

This scoping review was undertaken in line with Arksey and O’Malley’s framework for scoping studies [17] and reported according to Tricco and colleagues’ PRISMA extension for scoping reviews [18]. This purpose of this review was not to answer a specific and focused research question, or to critically appraise the available evidence, but rather to identify the available evidence on this topic, hence a scoping review was undertaken instead of a systematic review [19]. We included all primary studies that investigated the information needs and information seeking behaviour of people with dementia (irrespective of their stage of dementia) or their current non-professional caregivers. For the purpose of this review, we defined dementia as common dementia syndromes which include Alzheimer’s disease (AD), vascular dementia, presenile dementia, frontotemporal dementia syndromes (FTD), dementia with Lewy Bodies (LBD) and young onset dementia. Studies were also considered eligible for our review if the information needs or information seeking behaviour was based on the first-hand perspective of the person with dementia or their caregiver. We defined information needs as the need for new information, the need to confirm known information or the need to clarify known information [20]. We defined information seeking behaviour as “the purposive seeking of information as a consequence of a need to satisfy some goal”, following the definition by Wilson (pg 49) [21]. We only included studies in English.

We excluded studies that included patients with certain diagnoses (i.e. AIDS dementia complex, primary progressive aphasia, Creutzfeldt-Jakob syndrome, primary progressive non-fluent aphasia, Huntington disease, Kluver-Bucy syndrome), with comorbidities (e.g. dementia and Down’s syndrome), as the information needs of these patients and their caregivers may differ from those with more common dementia syndromes. We excluded studies that included patients without a definitive diagnosis of dementia, and caregivers of these patients. We excluded studies that explored the information needs of healthcare professionals as well as those focusing on general needs (such as service needs, psychological needs etc.) without mention of informational needs. We excluded studies that involved the evaluation of interventions for people with dementia and their non-professional caregivers.

### Data sources, collection, analysis

A comprehensive search of the literature was conducted across the following databases: Medline (Ovid), Embase (Ovid), PsycINFO (Ebsco) and CINAHL (Ebsco). We searched the databases in August 2018 for relevant studies. The search terms within our search strategy included: information seeking behaviour, help seeking behaviour, needs assessment, health service needs, information needs, caregivers, spouse caregivers, family caregivers, dementia and Alzheimer’s disease. We searched reference lists of related systematic reviews to ensure that relevant articles were not omitted. A search of grey literature, including various sites such as OpenGrey, Google and openDOAR was also performed (See Additional file 1).

The search results were imported into EndNote X8.2 [22] to form a single combined library. After duplicates were removed, two reviewers independently screened the collated titles and abstracts. Articles excluded during the title and abstract screening were not related to the topic. We did not prematurely exclude papers that seem to focus on the features or usefulness of information sources at the title and abstract screening stage, to avoid missing out on potentially relevant papers. The full texts of potential studies were retrieved and screened for their eligibility. Disagreements between the reviewers were resolved through discussion. The two reviewers independently extracted data using a data extraction form. Data for each study was extracted as follows:

- Study reference (author, year of publication, country where study was conducted, study design, study duration, study aims)
- Demographics of study population (number of participants, gender, age range, caregiver’s relationship to person with dementia, type/severity/duration of dementia, duration of caregiving, income and occupation, education level of caregiver, association with formal dementia care services)
- Types of information seeking behaviour
- Data measurement (data collection procedure, instrument used)
- Information needs identified
- Information sources (current, preferred sources of information)
- Other needs as specified by the person with dementia and/or caregiver

Inter-rater agreement between the two reviewers was calculated using Cohen’s kappa, and good agreement was determined to be achieved (kappa = 0.80) between the two reviewers. Discrepancies in the extracted data were resolved through discussion between the two reviewers. A third reviewer acted as a mediator where differences could not be resolved. Study authors were contacted where information was missing or incomplete.

No quality assessment was performed on the studies in this review, as the main aim of this scoping review was not to emphasise methodological inadequacies within the studies but to present a broad scope of the existing literature on the information needs and information seeking behaviour of people with dementia and their caregivers.

## Data synthesis and analysis

We performed a thematic analysis and categorised the data according to the emerging themes in the included studies. This was done independently by two reviewers in parallel. One reviewer generated the initial themes and the second reviewer applied this in parallel to the data. Discrepancies in thematic analysis were discussed between the study authors. For new themes that emerged subsequently, the two authors discussed to reach an agreement whether to include the new generated theme or reclassify it as part of an existing theme [23]. Categories were developed using a ‘bottom-up’ approach, and were refined throughout the review process. Within each category of information need mentioned, we listed the reported information needs according to frequency mentioned. By summarizing the data numerically, we endeavour to confirm and describe the patterns or regularities within information needs and information seeking behaviour [23]. Information needs were sorted into four main themes: dementia-specific information, patient care-related information, service information and caregiver self-care related information. We evaluated the frequency of information needs and other needs by calculating the percentage of studies that were included in each information need contributing to the main information theme [24, 25]. We also performed a secondary analysis of the data exploring the associations of different variables and information needs as well as the congruence between available and preferred information sources. We sought to evaluate if variables such as sociodemographic characteristics of the population and dementia severity associated with certain information needs and information seeking behaviour. Studies with similar themes were clustered together and the count and percentage of articles in each theme was calculated and reported [26].

We identified information seeking behaviour in the studies through patients’ and/or caregivers’ information sources, and their actions as specified in the article. For example, if a caregiver stated they often turned to their family doctor for more information on dementia, we would classify that as ‘active searching’ behaviour. Information seeking behaviour was grouped according to the four categories specified in Wilson’s model of information behaviour: active searching, passive searching, ongoing searching and passive attention [21]. Active searching was defined as the individual actively seeking out information (pg 3). Passive searching was defined as “the acquisition of information that happens to be relevant to the individual” (pg 3). Ongoing searching is “where active searching has already established the basic framework of knowledge, but where occasional continuing search is carried out to update or expand one’s framework” (pg 3). Passive attention is where “information acquisition may take place without intentional seeking” (pg 3) [27]. We selected Wilson’s model over other models of information behaviour as it offered a broad summary of the context of the information need of the seeker, the type of searching employed and the information sources utilised, and was not focused on the information seeking process. For current and preferred sources of information, the sources mentioned by studies in the literature were listed according to the frequency mentioned.

## Results

**Fig. 1:**
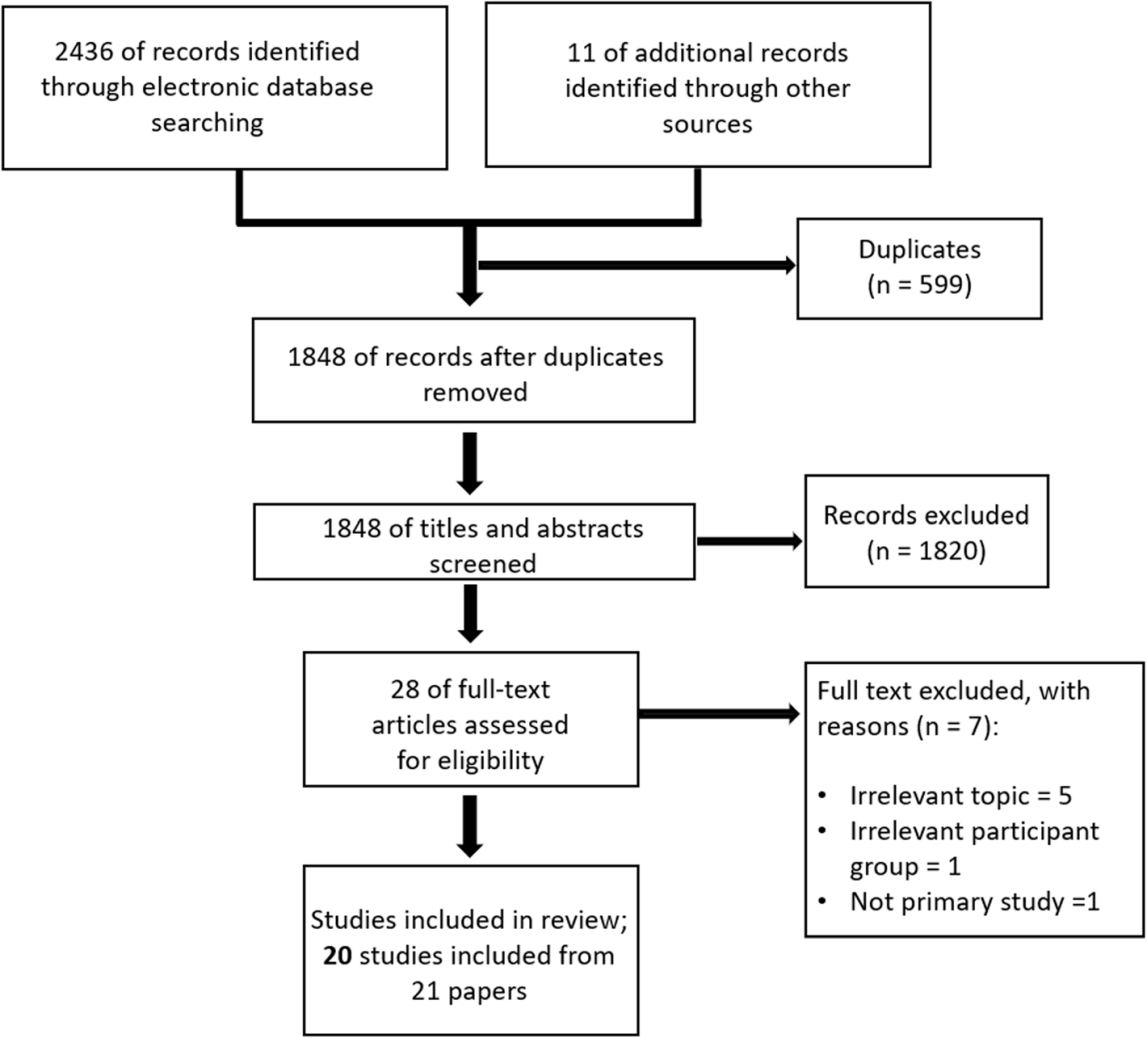
Study flow diagram

We identified a total of 2447 abstracts in our preliminary search. After duplicates were removed, we screened 1848 articles and eventually included 20 studies in this scoping study (Fig. 1). After screening for titles and abstracts, we retrieved the full texts for 38 articles that explored the information needs or information seeking behaviour of people with dementia and their caregivers. From these 38 articles, we excluded seven studies that did not meet our inclusion criteria. 20 studies from 21 reports, were eventually included in this review (Fig. 1).

The 20 studies comprised of ten cross-sectional studies, eight qualitative studies, and two mixed methods studies with 4140 participants. One of the cross-sectional studies was reported twice. All studies were presented in English and published between 2002 and 2018. Four studies examined both caregiver and patient perspectives while the rest examined only the caregiver’s. With the exception of four studies that did not report caregiver-patient relationship, the majority of the caregivers were the spouses or children of the person with dementia. All studies were from high income countries, with eight studies from the USA.Sample sizes ranged from 9 to 1181, apart from one study where sample size could not be determined due to study methodology. Majority of caregivers were female and had mean ages between 55 to 70 years. Mean ages of the person with dementia was between 70 to 90 years. Living arrangements of the person with dementia was reported in 9 out of the 20 studies, and most of the persons with dementia were community dwelling except in two studies where some of the persons with dementia received long-term care in nursing homes and institutions. There were also four studies that reported the use of dementia day care services by community dwelling persons with dementia. Education level of participants was stated in 11 studies, where most of the participants had completed at least high school education (Table 1).

**Table 1:** Characteristics of the included studies

Study, Year, Country	Characteristics							
	Study Design and Data Collection Methods	Participants	Gender^1^	Age^2^	Caregiver’s Relationship with Patient	Duration of Caregiving	Education Level of Caregiver	Study Objective
Boughtwood 2012 [36], Australia	Qualitative (Focus groups, Interviews)	121 family carers, 16 GPs, 20 geriatricians, 24 bilingual and bicultural workers	88 female, 33 male family carers	17–90	Not mentioned	Not mentioned	Not mentioned	To examine dementia-related information needs, access and delivery issues in Arabic, Chinese, Italian and Spanish-speaking communities in south-western Sydney, Australia
de Jong 2009 [45], Netherlands	Qualitative (Semi-structured interviews)	9 family carers	5 females, 4 male	Age range for persons with dementia: 67–88	4 spouse, 2 child, 1 daughter-in-law	Not mentioned	Not mentioned	To explore the needs and wishes of informal caregivers of people with dementia attending or formerly attending skilled psychogeriatric day-care in the Netherlands
Durcharme 2014, [28] Canada	Mixed methods (Interviews, Questionnaire)	32 family carers	24 female, 8 male	Mean: 54.28, SD: 10.5	25 spouse, 5 child, 2 other	Mean: 83.7 h per week, SD: 61.4 h	Mean: 14.34 years, SD: 3.2 years	To document the unmet support needs of early-onset dementia family caregivers
Edelman 2006, [46] USA	Cross sectional (22 item Checklist Survey)	100 family carers, 100 persons with dementia (caregivers paired with patients)	61 female, 39 male carers. 70 female and 30 male persons with dementia.	Mean: 61, SD: 13 for carers. Mean: 80, SD: 6 for persons with dementia	44 spouse, 46 parent/parent-in-law, 10 other relationship	Not mentioned	High school or less: 64, College education: 24, College graduates: 8, Graduate school: 4	To identify the information and service needs of persons with Alzheimer’s disease and their family caregivers living in rural communities and to assess differences and similarities in each partner’s perspective
Hirakawa 2009 [7], Japan	Cross sectional (Self-reported Structured Questionnaire)	475 family carers	366 female, 109 male	Mean: 64.9, SD: 12.2	205 spouse, 235 child (including daughter/son-in-law), 29 others	Not mentioned	Junior high education: 39, High School education: 200, University: 97	To explore the following two areas: (a) the priority information needs and sources of family caregivers of home elderly patients and (b) the differences in information needs according to the severity of dementia
Forbes 2012 [29], Canada	Qualitative (Interviews)	14 carers, 5 persons with dementia, 14 healthcare practitioners	11 female, 3 male carers. 2 female, 3 persons with dementia	Mean: 60.31, SD: 15.74 for carers. Mean: 77.40, SD: 11.67 for persons with dementia	6 spouse, 5 child, 2 grandchildren, 1 nephew	10 of the carers had provided care for 5 or more years, and 4 had provided care for less than 5 years.	Primary education or less: 1 Carer, Secondary education: 6 carers, Some post-secondary education: 1, Finished post-secondary education: 6	To enable healthcare practitioners, care partners and people with dementia to use dementia care and information more effectively by examining their information needs, how these change over time, and how they access, assess, and apply the knowledge
Galvin 2010 [34], USA	Cross sectional (Internet based survey)	971 carers	87.9% female, 12.1% male carers. 37.8% female, 62.2% male persons with dementia.	Mean: 55.9, SD: 12 for caregiver. Mean: 75.4, SD: 8.4 for persons with dementia.	40.6% spouse, 51.7% child, 3.8% other relative, 3.8% friend.	Not mentioned	Less than high school education: 0.6%, High school education: 25.5%, College education or higher: 73.8%	To ascertain the unmet needs of the Lewy Body dementia caregivers and collect data to inform educational programming and enhance caregiver support
Georges 2008 [37], Europe and UK	Cross sectional (Self-completed Questionnaire)	1181 carers	67% female, 33% male	65% ≥ 55 years of age, 33% < 55 years of age	47% spouse, 37% parent.	Not mentioned	Not mentioned	To identify carers’ needs, differences between countries with regard to dementia care and the level of satisfaction of carers with utilised services
Huis 2018 [30], Netherlands	Qualitative (Online focus groups)	36 family carers	32 female, 4 male	Mean: 61, SD: 42–81	19 child or child-in-law, 17 partner	Not mentioned	Primary school: 3, High school (preparatory to vocational education): 7, High school (preparatory to university education): 6, Applied/academic university: 16, Missing: 4	To discuss how and by whom family caregivers want to be supported in self-management when managing changes in behaviour and mood of relatives with dementia and whether family caregivers consider eHealth a useful tool for self-management support
Iribarren 2018 [38], USA	Qualitative (Participatory design session consisting of open dialogue, interactive feedback and browsing the internet, and a questionnaire)	24 carers	19 female, 5 male	Mean: 59.7, SD: 7.67	16 child, 6 spouse, 2 other relative	Mean: 6.5 years, SD: 4.7 years	Completed eight grade or less: 1, Completed all or some of high school: 12, Degree: 10	To identify caregiver attributes that may influences the use of Family Health Information Management System, caregiver information and communication needs and tasks, and caregiver perceptions of online tools to meet these needs
Jensen 2015 [39], USA	Mixed methods (Telephone survey, Online survey)	128 carers, 27 health care providers	76% female, 24% male carers	Mean: 62.5, SD: 12.5	47.6% child or child-in-law, 36.5% spouses.	3 years or more: 68.3%, Between 3 and 5 years: 38.9%, 8 or more years: nearly 15% (*N* = 17)	Completed some high school or had a high school degree: 17, Completed some college: 34, College degree: 33, Completed some graduate school/had a graduate degree: 33, Did not report education level: 11	To identify the needs of family caregivers and healthcare providers caring for persons with dementia, and characterize the needs of family caregivers, as they interact with the care recipient’s health care provider
Killen 2016 [35], UK	Cross sectional (Internet survey)	122 carers, 3 patients	89% female, 11% male	24 respondents were over 60, 101 respondents below 60	85 children, 22 spouses, 15 sisters or son/daughters-in-law or grandchildren	Not mentioned	Not mentioned	To explore the information and support needs of people with dementia with lewy bodies, and their caregivers around the point of diagnosis, in order to inform the development of theory-based, directly delivered interventions to improve coping with stress and increase quality of life
Koenig 2011 [31], USA	Cross sectional (48-item Survey)	33 dementia carers, 40 rehabilitation carers	29 female, 4 male dementia carers	Mean: 62.2	18 parent, 11 spouse, 3 other, 1 friend	Mean: 38.6 months	Mean 13.4 years of schooling.	To compare information needs of caregivers of persons with dementia with caregivers of those who received rehabilitation treatment
Rosa 2010 [32], Italy	Cross sectional (Questionnaire)	112 primary carers	77 female, 35 male	Mean: 55, SD: 10 for caregivers. Mean: 80, SD: 8 for persons with dementia	Not mentioned	Not mentioned	Not mentioned	To isolate the needs caregivers express within the following critical areas: medical, social, psychological and educational, to the effect of providing loved ones with support services capable of decreasing caregiving-related workloads
Scharett 2017 [40], USA	Cross sectional (Analysis of forum posts)	Alzheimer’s caregivers^3^	Not mentioned	Not mentioned	Not mentioned	Not mentioned	Not mentioned	To understand the characteristics of information caregivers of Alzheimer’s patients are searching for and the kind of support they receive through Internet-based peer support communities
Shreve 2016 [41], USA	Qualitative (Structured interviews)	12 family carers	11 female, 1 male	Not mentioned	Spouse, siblings, other relatives	Not mentioned	Not mentioned	To determine which information technology design characteristics and functionality family caregivers of adults with dementia would find most helpful
Thomas 2002 [33], France	Cross sectional (42-item Questionnaire)	408 caregiver/patient pairs	276 female, 126 male carers. 236 female, 172 male persons with dementia	Female caregivers: 60.66 ± 0.79, Male caregivers: 68.7 ± 1.13. Female patients: 77.1 ± 0.47, male patients: 75.7 ± 0.57	Not mentioned	Not mentioned	Not mentioned	To determine the complains of home caregivers, how they are interrelated and what causes them
Turner 2010, UK	Qualitative (Focus groups, interviews)	30 carers	18 female, 12 male	2/3 of carers were under 65 years of age	47% child, 37% spouse.	70% of carers had been carers for 2 years or more.	Not mentioned	To assess the needs for training of family carers of people with dementia, as part of an EU project to develop a training package for carers
Vaingankar 2013 [43], Singapore	Qualitative (Focus groups, semi-structured interviews)	63 informal carers	38 female, 25 male	Patients age range: 54 to 93	37 child, 13 spouse, 13 siblings/grandchildren/daughters or sisters-in-law	Not mentioned	None/some primary education: 5, Secondary/Junior college education: 10, Vocational education: 23, University education: 24	To elucidate the perceived unmet needs of informal caregivers of people with dementia in Singapore
Wackerbarth 2002 [44], USA	Cross sectional (Surveys)	128 carers	93 female, 35 male	Mean: 58.7, Range: 34–85 for caregivers. Mean: 78.5, Range: 53–96 for patients.	Children of patient (64.5%), spouse of patient (34.7%)	Mean: 4.7 years. Range 6 months to 8 years.	Mean of 14.6 years of education, range 8–22 years. Primary education only: 1.6%, Completed high school: 32%, Some college education: 62.8%.	To identify essential information and support needs of family caregivers for individuals with Alzheimer’s disease or a related dementia, and to examine the relationship between caregiver characteristics and needs

^1^ Gender refers to caregiver’s genders, unless otherwise specified

^2^ Age refers to caregiver’s age, unless otherwise specified

Of the types of dementia specified in this review, 35% of studies included dementia [7, 28–33], 20% of studies included LBD [30, 33–35], 15% of studies included AD and other non-specific forms of dementia [28, 30, 33]. However, 45% of studies did not specify the type of dementia [36–44]. All 20 studies identified information needs irrespective of dementia types, however information seeking behaviour was only reported in 11 studies. Four studies further illustrated barriers to information needs and information seeking behaviour which included: lack of access, time, energy, knowledge to interpret or search for information and denial of the condition [7, 28, 29, 36].

### Information needs

We identified four main themes in relation to information needs, with a total of 39 reported information needs. The four information themes were disease-specific information, healthcare service-related information, patient care provision, and caregiver self-care. Information related to healthcare services was the most commonly identified theme, while patient care provision represented the category with the most diverse number of information needs.

#### Disease-specific information

The three most commonly mentioned needs related to dementia were general information on dementia (10 studies, 62.5%) [32–35, 38–40, 42, 44, 45], dementia treatment (7 studies, 43.7%) [7, 28, 29, 34, 43, 44, 46] and identification and understanding of dementia symptoms and behaviour (6 studies, 37.5%) [7, 35, 40, 42, 43, 46]. Other information needs within this category also included dementia prognosis (6 studies, 37.5%) [7, 29, 33, 37, 39, 41], current dementia medication (5 studies, 31.2%) [32, 35, 38, 40, 46], experimental drugs and clinical trials (4 studies, 25%), current research on dementia (3 studies, 18.7%) [34, 35, 44] and genetic aspects of dementia (2 studies, 12.5%) [35, 46]. The following information needs were mentioned only once (0.06%): negative impact of dementia on family and community [7], information specific to different stages of dementia [43], chance of recovery [7] and memory skills [35] (Table 2).

**Table 2:** Information needs themes of caregivers and dementia patients (*n* = 20 articles)

Theme	Information need	Number and percentage of studies^a^	References for information need
Disease-specific information	General information on dementia	10 (62.5%)	[32–35, 38–40, 42, 44, 45]
	Dementia Treatment	7 (43.7%)	[7, 28, 29, 34, 43, 44, 46]
	Identifying and understanding dementia eg. typical symptoms of dementia/behaviour	6 (37.5%)	[7, 35, 40, 42, 43, 46]
	Dementia prognosis	6 (37.5%)	[7, 29, 33, 37, 39, 41]
	Current dementia medication	5 (31.2%)	[32, 35, 38, 40, 46]
	Experimental drugs/Clinical trials	4 (25%)	[29, 34, 44, 46]
	Current research on dementia	3 (18.7%)	[34, 35, 44]
	Genetic aspects of the disease	2 (12.5%)	[35, 46]
	Negative impact of dementia on family and community	1 (0.06%)	[7]
	Information specific to different stages of dementia	1 (0.06%)	[43]
	Chance of recovery	1 (0.06%)	[7]
	Memory Skills	1 (0.06%)	[35]
Patient Care Provision information	How to care for the patient eg. general care, patient hygiene, food and nutritional information, best attitudes to adopt in caring	7 (53.8%)	[30–32, 35, 36, 45, 46]
	How to deal with patient’s behaviour	6 (46.1%)	[30–32, 35, 36, 45]
	Safety issues eg. how to improve safety of environment, how to keep patient safe, how to recognise fall risks and poor mobility	5 (38.4%)	[28, 29, 35, 40, 45]
	Coping with hallucinations	2 (15.3%)	[31, 35]
	Communication difficulties and how to manage them	2 (15.3%)	[35, 46]
	Patient activities	2 (15.3%)	[38, 46]
	First aid and medical information	2 (15.3%)	[7, 45]
	Emergency situations	2 (15.3%)	[28, 43]
	Conflict resolution	1 (0.07%)	[40]
	Patient Ethics	1 (0.07%)	[40]
	Helpful experiences of other caregivers	1 (0.07%)	[35]
	How to deal with family and friends	1 (0.07%)	[46]
	How to advocate for patient	1 (0.07%)	[29]
	When to transfer patient to hospital	1 (0.07%)	[7]
Healthcare Service-related	Where and how to use services/help available e.g. geriatric hospitals, nursing homes, support groups, physicians skilled in diagnosis and treatment	14 (93.3%)	[7, 28, 29, 32, 34–37, 39, 40, 42–44, 46]
	Financial help and services	5 (33.3%)	[28, 39, 40, 42, 44]
	Legal issues	4 (26.6%)	[7, 29, 40, 44]
	How to apply for care programs eg. day care, long term care	2 (13.3%)	[29, 32]
	Insurance	2 (13.3%)	[38, 44]
	Home Help	1 (0.07%)	[33]
	Transportation options	1 (0.07%)	[29]
Caregiver self-care	Stress Management	2 (50%)	[7, 31]
	Carer’s entitlements (pension)	1 (25%)	[36]
	Managing emotions	1 (25%)	[31]
	General caregiver self-care eg. exercise, diet, own medications	1 (25%)	[38]

^**a**^Percentage of studies refer to the percentage of studies in each reported information need contributing towards the main theme

The need for information on negative impact of dementia on family and community was defined by Hirakawa as the physical and psychosocial burden on family and community [7]. It was stated that this would help the caregivers prepare for and cope with the behavioural and psychological symptoms of dementia, as these symptoms worsen through the progression of the disease.

#### Patient care provision

Patient care provision was the category with the largest number of reported information needs, comprising 14 out of the 39 unique information needs (35.8%) identified in this review. Seven studies (53.8%) within this category indicated a need for information on how to provide general care [30–32, 35, 36, 45, 46]. General care included patient hygiene, food and nutritional information and best attitudes to adopt in caring. Ways to deal with the patient’s behaviour (6 studies, 46.1%) was the second most commonly mentioned information need [30–32, 35, 36, 45], followed by safety issues (5 studies, 38.4%) [28, 29, 35, 40, 45]. Safety issues encompassed information on how to improve the safety of the patient’s home environment, how to keep patient safe and how to recognise fall risks and poor mobility. Other reported information needs in this category were coping on with patient hallucinations (2 studies, 15.3%) [31, 35], navigation of communication difficulties (2 studies, 15.3%) [35, 46], activities for the patient (2 studies, 15.3%) [38, 46], first aid and medical information (2 studies, 15.3%) [7, 45] and emergency situations (2 studies, 15.3%) [28, 43]. Additional seven information needs were retrieved from only one study and these were: conflict resolution [40], patient ethics [40], helpful experiences of other caregivers [35], when to transfer the patient to the hospital [7], dealing with family and friends [46] and advocating for the patient [29].

#### Healthcare service-related

The top information need within this category was where and how to use services, and available help (14 studies, 93.3%) [7, 28, 29, 32, 34–37, 39, 40, 42–44, 46]. This was also the information need that was reported by the largest number of studies, among all identified needs. Services and help referred to facilities (e.g. geriatric hospitals and nursing homes), healthcare professionals skilled in dementia diagnosis and treatment, or support groups for caregivers. Other service-related information needs were financial help and services (5 studies, 33.3%) [28, 39, 40, 42, 44], legal issues (4 studies, 26.6%) [7, 29, 40, 44], application for care programs (2 studies, 13.3%) [29, 32], and insurance (2 studies, 13.3%) [38, 44]. As with the other main information themes, there were several information needs that were only stated once (0.07%). For service-related information, this included home help [33], and transportation options [29].

#### Caregiver self-care

Self-care for the caregiver was only mentioned by four studies [7, 31, 36, 38]. Two studies identified information needs for stress management (50%) [7, 31], while carer’s pension entitlements [36], managing caregiver’s emotions [31], and general caregiver self-care such as exercise, diet and medications [38] were identified by one study each (25%). This information theme had the smallest number of contributing studies and reported information needs.

### Variables linked to information needs

The severity of dementia and patient/caregiver status were the only variables found to be potentially linked to particular information needs in this review.

#### Information needs at various stages of dementia

Caregivers of people with mild dementia were more likely to look for disease-specific information, as compared to caregivers of people with moderate to severe dementia. Both groups of caregivers required general information on dementia, identifying and understanding dementia and current dementia medication. However, those caring for people with mild dementia also needed information on genetic aspects of the disease, experimental drugs and current research on dementia while those caring for people with advanced dementia were primarily concerned about dementia prognosis (Table 3).

**Table 3:** Information needs classified according to severity of dementia

Author^(reference)^, year	Severity of dementia	Information needs
Edelman [46], 2006	Mostly mild	1. Identifying and understanding dementia 2. Experimental drugs 3. Current dementia medication 4. Genetic aspects of the disease 5. Current research on dementia 6. Where and how to use services/help available
Hirakawa [7], 2011	Mostly mild	1. General information on dementia 2. First aid and medical information 3. How to care for the patient 4. When to transfer patient to hospital 5. How to apply for care programs
de Jong [45], 2009	Mostly moderate - severe	1. General information on dementia 2. Identifying and understanding dementia 3. Dementia prognosis 4. How to deal with patient’s behaviour 5. Safety issues 6. Medical information 7. Where and how to use services/help available
Rosa [32], 2010	Mostly moderate - severe	1. General information on dementia 2. Identifying and understanding dementia 3. Current dementia medication 4. How to deal with patient’s behaviour 5. How to apply for care programs 6. Where and how to use services/help available 7. Financial help and services

The severity of the patient’s condition did not affect the number of patient care provision-related information needs. While both groups of caregivers had equal numbers of patient care information needs, the subcategories of information requested did not overlap. Caregivers of people with mild dementia needed information on how to care for the patient, first aid and when to transfer a patient to the hospital. Caregivers of people with moderate to severe dementia desired medical information, information on how to deal with patient’s behaviour and safety issues.

Carers of people with moderate to severe dementia also needed more information on healthcare services. Both groups of carers required information on where and how to use available services, and how to apply for daycare programs. Caregivers of patients in more advanced stages of dementia indicated an additional need for information on financial help and services.

#### Patient versus caregiver information needs

Three studies investigated the needs of people with dementia alongside their caregivers [33, 35, 46]. However, two of these studies did not segregate the information needs of people with dementia from caregivers, therefore we were unable to present any differences in information needs for these particular studies [33, 35].

The remaining study, that did segregate the information needs of people with dementia and their caregivers, showed that people with dementia and caregivers had a large overlap in 80% of their information needs [46]. The topics of shared interest included identifying and understanding dementia, experimental drugs and clinical trials, current dementia medication, communication difficulties and how to manage them, how to deal with patient’s behaviour, and patient activities. These topics were equally distributed between disease-specific and patient care provision themes. The topics of interest specific to the caregivers were genetic aspects of the disease, and how to deal with family and friends. On the other hand, the topics of interest unique to people with dementia were clinical trials and where to find support groups. Overall, caregivers were more interested in topics specific to dementia, while people with dementia were more concerned about service-related topics such as where to find support groups for those with memory loss.

### Information seeking behaviour

We classified information seeking behaviour into four categories, following Wilson’s model of information behaviour [21]. The four categories were passive attention, passive searching, active searching and ongoing searching. Information seeking behaviour was illustrated in 12 out of 20 studies [7, 29, 30, 33–36, 38–40, 43, 46]. Ten studies reported active searching behaviour [29, 30, 33, 34, 36, 38–40, 43, 46], such as when a caregiver or person with dementia actively approaches a healthcare professional for more information on their condition, or attempts to retrieve relevant information by themselves. Four studies showed passive attention behaviour [7, 33, 35, 43], where information is obtained from the environment such as when the radio or television is turned on, without the intention of information seeking. Ongoing searching was indicated in one study, where participants continued their search for information by attending seminars or facilitated forums, to expand their knowledge on the condition and its management [36]. Passive searching behaviour was indicated correspondingly in one study, in which a regular behaviour such as reading of newspapers/magazines enables the person with dementia and/or their caregiver to obtain relevant information [7]. Four studies gave examples of two types of information seeking behaviour [7, 33, 36, 43]. Two of these studies feature contrasting information searching behaviours of active searching and passive attention [33, 43]. The other two studies had similar types of information seeking behaviours and were only active (active searching, ongoing searching) [36] or passive behaviours (passive attention, passive searching) [7].

Information seeking behaviour was mainly surmised from current information sources stated within the studies. These sources for patients and/or their caregivers were stated in 14 studies [7, 28–30, 33, 35, 36, 38–41, 43, 44, 46]. Of these 14 studies, six studies also mentioned desired information sources [30, 35, 36, 38, 41, 43]. Two studies mentioned the preferred sources of caregivers without reference to their current information sources [34, 42]. The most utilised source of information was the Internet (e.g. websites and forums) (10 studies, 71.4%) [7, 28–30, 36, 38–41, 46], followed by healthcare professionals (7 studies, 50%) [7, 29, 30, 35, 39, 43, 44], and family and friends (6 studies, 42.8%) [7, 29, 30, 35, 36, 44]. The least common sources of information were bank employees [29] and clergy [44], as they were mentioned in only one study each. Other sources of information utilised include the Alzheimer’s Association, written information sources (eg. magazines, leaflets and books), electronic sources (e.g. email, smartphone apps, videos), support groups, care centres, social workers, other family caregivers, and volunteer groups (Table 4).

**Table 4:** Current and preferred information sources on dementia in the included studies

	Information Sources	Number and percentage of studies	References for each source
Current sources	Internet - Websites and forums	10 (71.4%)	[7, 28–30, 36, 38–41, 46]
	Healthcare professionals	7 (50%)	[7, 29, 30, 35, 39, 43, 44]
	Family/friends	6 (42.8%)	[7, 29, 30, 35, 36, 44]
	Alzheimer’s Association website	5 (35.7%)	[29, 30, 36, 39, 44]
	Electronic sources – email, smartphone apps (eg. Alzheimer’s Assistant), videos, TV	5 (35.7%)	[30, 33, 38, 40, 43]
	Written information - books, leaflets, newsletters, newspapers, magazines	4 (28.5%)	[7, 33, 39, 40]
	Support groups	4 (28.5%)	[29, 38, 39, 44]
	Care centres	2 (14.2%)	[29, 44]
	Social worker	2 (14.2%)	[35, 44]
	Other family caregivers	2 (14.2%)	[30, 43]
	Volunteer groups/organizations	2 (14.2%)	[7, 35]
	Clergy	1 (0.07%)	[44]
	Bank employees	1 (0.07%)	[29]
Preferred sources	Internet - Website page with FAQs, how-to videos, online portals	4 (66.6%)	[30, 38, 41, 43]
	Electronic sources – mass media, audio-visual materials, DVDs, smartphones	4 (66.6%)	[34, 36, 41, 43]
	Support groups	3 (50%)	[34, 35, 42]
	Written information	3 (50%)	[34, 42, 43]
	Electronic medical records (EMR)	1 (16.6%)	[38]
	Educational conferences	1 (16.6%)	[34]
	Government services	1 (16.6%)	[36]
	Healthcare professionals	1 (16.6%)	[36]

**Fig. 2:**
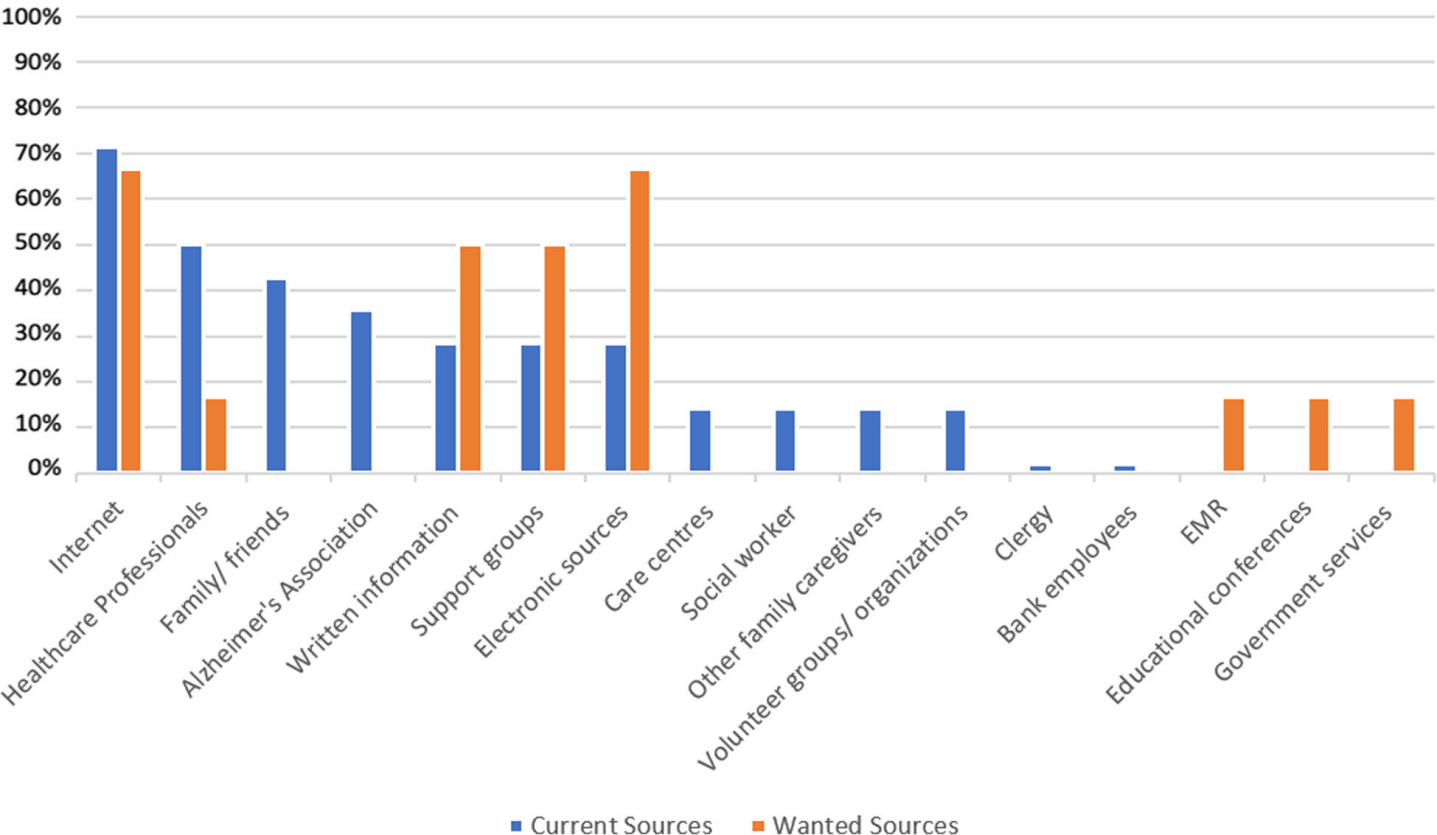
Percentage of Studies indicating Current and Preferred Information Sources

The Internet was indicated as both the most highly utilised source of information and the most desired source of information for persons with dementia and non-professional caregivers. Most of the persons with dementia and their caregivers used the Internet by browsing websites and forums to look for information. However, what people with dementia and caregivers desired when using the Internet were more specific online resources, such as a website with frequently asked questions, how-to videos for patient care and service-related information available in online portals [30, 38, 43]. Unsurprisingly, other electronic sources such as mass media, smartphones, audio-visual materials and DVDs ranked equally as high as the Internet, as a preferred source of information [34, 36, 41, 43]. The electronic sources requested were not dissimilar to the current electronic sources already utilised by people with dementia and their informal caregivers. Persons with dementia and caregivers showed equal preference for written information sources and both online and face-to-face support groups (50%), despite those sources not being one of the top three current information sources [34, 35, 42, 43]. Electronic medical records [38], educational conferences [34], government services [36] and healthcare professionals [36] were also reported as desired information sources, in one study each (Table 4, Fig. 2).

In addition to the abovementioned sources, one study also stated additional preferences on how information should be presented [43]. Having information in multiple sources and languages was preferred, in order to increase its accessibility to linguistically diverse communities. Information presented through these sources should also be individualised to the different dementia syndromes and stages of the disease, as the concerns of these patients and caregivers could vary widely.

## Discussion

We identified 20 studies (4140 participants) reporting the information needs and information seeking behaviour of people with dementia and their non-professional caregivers. Studies mostly focused on the information needs and information seeking behaviour of dementia caregivers, were conducted in high income countries and involved predominantly female caregivers. Most of the persons with dementia were community dwelling, with a small proportion of persons with dementia in long term institutional care. Dementia health services related information was the most commonly reported information need in the included studies. The most diverse information needs related to patient care. Severity of dementia and patient/caregiver status were the only variables observed to be associated to information needs of dementia caregivers and persons with dementia. Information seeking behaviour was reported in 14 studies, and active searching behaviour was the most commonly reported type of information behaviour. Currently, there is a lack of evidence focused on the information needs and information seeking of people with dementia, and patients with different types of dementia. More research is also required for populations in low- and middle-income countries, and for comparison of the impact of different variables on information needs and information seeking behaviour.

The results of this current review suggest that the type of information most often requested by people with dementia and their informal caregivers was where to find and use available dementia-focused healthcare services (14 out of 20 studies; Table 2). Surprisingly, this contradicted most of the available literature that we have seen. Systematic reviews exploring the needs of informal dementia care providers indicated that the most frequently reported information need was information on the disease, followed by patient care information [8, 47]. There could be several reasons for this inconsistency. We included several studies that were published after the dates of these reviews. As such, studies that were more recently published could have implied there was a shift in the focus of information needs away from the disease and towards available help and services. At the point of diagnosis, the persons with dementia and their caregivers could have been counselled on the condition to help them understand and accept the condition. However, information about formal care services may not always be adequately provided at the point of diagnosis, and this information becomes more pertinent as the patient’s condition declines [48]. As seen in our results, the severity of the patient’s dementia condition also affects the type of information required by their caregivers and the person with dementia themselves. While we are unable to establish if this was a definite contributor to the difference in information needs as both reviews did not provide the stages of dementia for their included studies [8, 47], this could have also influenced the information needs reported as caregivers usually need more help in the later stages of dementia, following the worsening of behavioural and psychological symptoms of dementia. Another possible reason would be that the focus of these systematic reviews was not on information needs, but rather on general needs, and thus did not include some of the studies and information needs that were mentioned in the current review.

In order to better understand the information preferences of persons with dementia and their informal caregivers, we also evaluated their information seeking behaviour and information sources utilised. It was reported that the most desired source of information was the one that patients and caregivers most frequently utilised (i.e. the Internet). This suggests that their information needs were unmet while using the Internet, as a result of the inability of these groups to find the information they need or the inadequacy of information available on specific websites or portals that were accessed. Our results seem to suggest that the latter was a more likely reason, as participants indicated specific resources they wished to find on the Internet, such as videos and frequently asked questions (FAQs) websites (see Table 4). The sentiment on being unable to find the specific types of information they were looking for across various information sources has also been echoed in existing literature [8]. The inability to find specific information may not be due to a lack of information, but in certain cases it could also be presented in a form that was difficult to understand by people with dementia and their caregivers. Information on the disease could have been presented using medical terms that people were not familiar with. Often, the amount of information available could be overwhelming for people with dementia and their care providers to sort through, leading them to miss what they were looking for even if it was readily available. Despite only one study in our review emphasising the need for targeted and timely information to people with dementia and their caregivers, this need is not unique based on types and severity of dementia. This was also previously iterated in two reviews [8, 49]. Future interventions focusing on information provision should take these into account when developing information resources.

An interesting finding from our review was that the top sources of information that people with dementia and caregivers preferred were electronic sources such as the Internet, mass media and smartphones. This was unexpected given that the mean ages of caregivers were between 55 to 70 years, and between 70 to 90 years for the people with dementia. Existing literature to date suggests that electronic sources were not preferred as health information sources due to a lack of awareness about what these sources could offer, rather than cost or access-related reasons [50]. As internet adoption among older adults have steadily increased over the years, the purposes for which they used the Internet for could also have changed. In 2003, the use of the Internet in older adults were mainly confined to email and general information seeking but more recent analyses in 2009 show that in addition to those purposes, health information seeking was placed as one of the more common online activities [51, 52]. Electronic sources are able to offer a variety of information independent of time and location, which could be the reason for their growing popularity as a health information source. This growing trend of information retrieval from electronic sources is not limited to the Internet but could also encompass apps for dementia care on smartphones and programmes on mass media sources [53, 54]. The living arrangements of persons with dementia would affect the caregiving roles of their informal carers, and subsequently their information needs. However, we did not find any differences between the needs of carers of community dwelling persons with dementia compared to needs of carers of persons with dementia receiving long-term institutional care. Our results also identified active searching behaviour as the most common information searching behaviour amongst all included studies, highlighting the growing acceptance of new information sources (such as smartphones and the Internet) that were previously not used by persons with dementia and caregivers.

People with dementia and caregivers also showed a preference for written information and support groups as information sources over healthcare professionals. Healthcare professionals were usually the first information source that persons with dementia and caregivers would encounter, with information about the condition being provided at point of diagnosis. However, several studies within our review expressed that the information provided was insufficient and that further information was also not provided at subsequent follow up appointments [30, 36, 39, 42]. The information provided by healthcare professionals could be of a medical nature (such as medications to manage the condition), while persons with dementia and caregivers may desire information regarding the prognosis of the condition or available community care resources and entitlements. This finding again highlights the mismatch between information needs and information sources, where healthcare professionals may not necessarily be unable to provide the information required but could be unaware of these other information needs of the patients and caregivers. Support groups and written information sources could also be more highly preferred due to their ability to provide an array of information beyond medical information.

### Limitations and future research directions

Our review identified several limitations. We were unable to ascertain the severity of dementia for the persons with dementia, and if the participants included in some studies were truly the primary caregivers for persons with dementia, as some surveys were returned through the post or online. While we did not set restrictions on the geographical regions of where studies were conducted, all studies were conducted in high income countries such as the USA and UK, therefore the findings may not be applicable to other geographical regions or even rural areas within these countries. We recognize that there is a high prevalence of comorbidities in people with dementia, however the findings from this review may not be generalizable to persons with dementia and other chronic conditions or comorbidities, as studies involving these populations were excluded as part of our exclusion criteria. Furthermore, due to the small number of studies within this review, the findings on the associations between different variables and information needs should only be seen as a hypothesis generating. Our search strategy was limited to studies published in English and we may have missed relevant articles that were published in other languages. Most of the studies included addressed mainly the information needs and information seeking behaviour of the caregivers, and only four studies addressed the needs of both people with dementia and caregivers.

Some studies recruited participants through self-selection, therefore they may already be more highly educated, have higher income levels and increased informational needs and already be more active in searching for information.

Our scoping review highlights that the mismatch between information needs and information sources is not due to access, but rather the inability to find specific information across various sources. Rather than finding new ways and platforms to deliver information to patients and their caregivers, which would result in further fragmentation of available information, effort should be made to consolidate information on specific sites or platforms that are already in use (e.g. Alzheimer’s Association website). Our findings point towards a need for a change in clinical practice. Healthcare services should ensure access to clear, understandable and helpful sources that address patients’ and caregivers’ needs presented in this review. In addition, clinicians can help address these information needs by proactively inquiring about them and recommending relevant and helpful resources to persons with dementia and their family. Future interventions targeted at information provision should focus on utilising familiar platforms to deliver information pertaining to healthcare services while ensuring that the information is available in different languages.

Most of the studies included in the review used mainly cross-sectional [7, 9, 31–35, 37, 40, 44, 46] or qualitative study designs [29, 30, 36, 38, 41–43, 45]. The limited observational evidence found in this review provides preliminary findings on the associations between different factors and needs in some settings, and these findings should be further explored in future within larger, well-designed studies. Future research should consider the use of longitudinal study designs, in order to further identify information needs of patients and caregivers at various stages of their disease – from diagnosis where disease related information may be more desired, to the more advanced stages where help may be sought on providing adequate care to the patient. With the advances in technologies, future research should explore how information can be delivered in a targeted manner based on different types of digital technologies, such as through smartphones. The use of mobile phone technologies for health professions education has seen great effectiveness and is also a cost-effective and accessible alternative to printed information sources [55]. Since there is already an indicated preference for electronic sources, this approach could similarly be adapted to deliver information to people with dementia and caregivers.

## Conclusion

This scoping review provides an overview of the literature on the information needs and information seeking behaviour of people with dementia and caregivers. Information needs of people with dementia and caregivers can be categorised into four themes – disease related information, healthcare services-related information, patient care provision and caregiver self-care. Information on healthcare services need to be emphasised by healthcare professionals at consultations and through preferred electronic and written sources. Caregiver self-care was also another information need that was frequently overlooked, and future research should focus on evaluation of this information need. The only variables found to be associated to information needs were the severity of dementia as well as patient/caregiver status. Information needs between caregivers and persons with dementia were found to be largely consistent, however, more research is required on this area as this was only highlighted by one study. People with dementia and caregivers actively sought for information and preferred using the Internet to look for desired information. Future interventions on information delivery to people with dementia and caregivers should focus on having required information on central platforms such as the Alzheimer’s Association website and emphasise the use of preferred electronic sources. Future research should aim to evaluate the information needs of patients and caregivers at specific stages, across various dementia syndromes and focus on variables that affect these information needs.

## Supplementary information

**Additional file 1.** Search strategy.

## Data Availability

This scoping review included the data extracted from the primary studies. The whole set of data extraction sheet is available upon request from the corresponding author.

## Abbreviations

AD: Alzheimer’s disease
DVD: Digital versatile disc
EMR: Electronic medical records
EU: European Union
FAQs: Frequently asked questions
FTD: Frontotemporal dementia
GP: General practitioner
LBD: Lewy Body dementia
