# Supplementary material for: Information needs and information seeking behaviour of people with dementia and their non-professional caregivers: a scoping review
Source: BMC Geriatr. 2020 Feb 14;20:61. doi: 10.1186/s12877-020-1454-y (PMC7023704; doi:10.1186/s12877-020-1454-y)
Supplement: Supplementary file 1 — Additional file 1. Search strategy. [file 12877_2020_1454_MOESM1_ESM.docx]

**Additional file 1. Search strategy**

CINAHL (EBSCOhost)

Updated 17/08/18 10:53

| S# | Search Terms | Search options | Results |
| --- | --- | --- | --- |
| 1 | MH Information Seeking Behavior+ | **Search modes** - Find all my search  terms | 3,426 |
| 2 | TI (Information Seek* or information-seek*  behavio#r) OR AB (Information Seek* or information- seek* behavio#r) | **Search modes** -  Find all my search terms | 8,796 |
| 3 | MH Help Seeking Behavior+ | **Search modes** -  Find all my search terms | 5,120 |
| 4 | TI (Help Seek* or help seek* behavio#r) OR AB (Help Seek* or help seek* behavio#r) | **Search modes** - Find all my search terms | 8,537 |
| 5 | TI Information need* OR AB Information need* | **Search modes** -  Find all my search terms | 3,050 |
| 6 | TI Health services N3 need* OR AB Health services N3 need* | **Search modes** - Find all my search  terms | 12,142 |
| 7 | S1 OR S2 OR S3 OR S4 OR S5 OR S6 | **Search modes** -  Find all my search terms | 54,356 |
| 8 | MH Caregivers+ | **Search modes** -  Find all my search terms | 28,335 |
| 9 | TI ("Care giv*" or "carer*" or "family caregiv*" or "spouse caregiv*") OR AB ("Care giv*" or "carer*" or "family caregiv*" or "spouse caregiv*") | **Search modes** - Find all my search terms | 20,096 |
| 10 | TI patient* OR AB patient* | **Search modes** -  Find all my search terms |  |
| 11 | S8 OR S9 OR S10 | **Search modes** - Find all my search  terms | 40,379 |
| 12 | MH Dementia+ | **Search modes** -  Find all my search terms | 59,523 |
| 13 | TI (Dementia OR Amentia or "Familial Dementia" or "Senile Paranoid Dementia") OR AB (Dementia OR Amentia or "Familial Dementia" or "Senile Paranoid  Dementia") | **Search modes** - Find all my search terms | 39,487 |
| 14 | MH "Alzheimer's Disease+" | **Search modes** -  Find all my search terms | 26,425 |
| 15 | TI ("Alzheimer Disease" or "Acute Confusional Senile Dementia" or "Alzheimer Dementia" or "Alzheimer Disease, Early Onset" or "Alzheimer Disease, Late Onset" or "Alzheimer Sclerosis" or "Alzheimer Syndrome" or "Alzheimer Type Senile Dementia" or  "Alzheimer's Disease" or "Alzheimer's Disease, Focal Onset" or "Alzheimer-Type Dementia (ATD)" or | **Search modes** - Find all my search terms | 22,738 |

|  | "Dementia, Alzheimer Type" or "Dementia, Presenile" or "Dementia, Primary Senile Degenerative" or "Dementia, Senile" or "Early Onset Alzheimer Disease" or "Familial Alzheimer Disease (FAD)" or "Focal Onset Alzheimer's Disease" or "Late Onset Alzheimer Disease" or "Presenile Alzheimer Dementia" or "Primary Senile Degenerative Dementia" or "Senile Dementia, Acute Confusional" or "Senile Dementia, Alzheimer Type" ) OR AB  ( "Alzheimer Disease" or "Acute Confusional Senile Dementia" or "Alzheimer Dementia" or "Alzheimer Disease, Early Onset" or "Alzheimer Disease, Late Onset" or "Alzheimer Sclerosis" or "Alzheimer Syndrome" or "Alzheimer Type Senile Dementia" or "Alzheimer's Disease" or "Alzheimer's Disease, Focal Onset" or "Alzheimer-Type Dementia (ATD)" or "Dementia, Alzheimer Type" or "Dementia, Presenile" or "Dementia, Primary Senile Degenerative" or "Dementia, Senile" or "Early Onset Alzheimer Disease" or "Familial Alzheimer Disease (FAD)" or "Focal Onset Alzheimer's Disease" or "Late Onset Alzheimer Disease" or "Presenile Alzheimer Dementia" or "Primary Senile Degenerative Dementia" or "Senile Dementia, Acute Confusional" or  "Senile Dementia, Alzheimer Type") |  |  |
| --- | --- | --- | --- |
| 16 | MH "Dementia, Vascular+" | **Search modes** - Find all my search  terms | 1,342 |
| 17 | TI ( "Dementia, Vascular" or "Acute Onset Vascular Dementia" or "Arteriosclerotic Dementia" or "Arteriosclerotic Encephalopathy, Subcortical" or "Binswanger Disease" or "Binswanger Encephalopathy" or "Binswanger's Disease" or "Chronic Progressive Subcortical Encephalopathy" or "Encephalopathy, Binswanger" or "Encephalopathy, Binswanger's" or "Encephalopathy, Chronic Progressive Subcortical" or "Encephalopathy, Subcortical Arteriosclerotic" or "Encephalopathy, Subcortical, Chronic Progressive" or "Leukoencephalopathy, Subcortical" or "Subcortical Arteriosclerotic Encephalopathy" or "Subcortical Encephalopathy, Chronic Progressive" or "Subcortical Leukoencephalopathy" or "Subcortical Vascular Dementia" or "Vascular Dementia" or "Vascular Dementia, Acute Onset" ) OR AB ( "Dementia, Vascular" or "Acute Onset Vascular Dementia" or "Arteriosclerotic Dementia" or "Arteriosclerotic Encephalopathy, Subcortical" or "Binswanger Disease" or "Binswanger Encephalopathy" or "Binswanger's Disease" or "Chronic Progressive Subcortical Encephalopathy" or "Encephalopathy, Binswanger" or "Encephalopathy, Binswanger's" or "Encephalopathy, Chronic Progressive Subcortical" or "Encephalopathy, Subcortical Arteriosclerotic" or  "Encephalopathy, Subcortical, Chronic Progressive" | **Search modes** - Find all my search terms | 1,452 |

|  | or "Leukoencephalopathy, Subcortical" or "Subcortical Arteriosclerotic Encephalopathy" or "Subcortical Encephalopathy, Chronic Progressive" or "Subcortical Leukoencephalopathy" or "Subcortical Vascular Dementia" or "Vascular Dementia" or "Vascular  Dementia, Acute Onset") |  |  |
| --- | --- | --- | --- |
| 18 | MH "CADASIL+" | **Search modes** - Find all my search terms | 192 |
| 19 | TI (CADASIL or CADASILM or "Cerebral Arteriopathy with Subcortical Infarcts and Leukoencephalopathy" or "Cerebral Autosomal Dominant Arteriopathy with Subcortical Infarcts and Leukoencephalopathy" or "Dementia, Hereditary Multi-Infarct Type") OR AB  (CADASIL or CADASILM or "Cerebral Arteriopathy with Subcortical Infarcts and Leukoencephalopathy" or "Cerebral Autosomal Dominant Arteriopathy with Subcortical Infarcts and Leukoencephalopathy" or  "Dementia, Hereditary Multi-Infarct Type") | **Search modes** - Find all my search terms | 270 |
| 20 | MH "Dementia, Multi-Infarct+" | **Search modes** - Find all my search  terms | 263 |
| 21 | TI ("Dementia, Multi-Infarct" or "Dementia Multi- Infarct" or "Dementia, Multiinfarct" or "Lacunar Dementia" or "Lacunar Dementias" or "Multi-Infarct Dementia" ) OR AB ( "Dementia, Multi-Infarct" or "Dementia Multi-Infarct" or "Dementia, Multiinfarct" or "Lacunar Dementia" or "Lacunar Dementias" or "Multi- Infarct Dementia" ) OR TX ( "Dementia, Multi-Infarct" or "Dementia Multi-Infarct" or "Dementia, Multiinfarct" or "Lacunar Dementia" or "Lacunar Dementias" or  "Multi-Infarct Dementia") | **Search modes** - Find all my search terms | 713 |
| 22 | MH "Diffuse Neurofibrillary Tangles with Calcification+" | **Search modes** - Find all my search  terms | 492 |
| 23 | TI ("Diffuse Neurofibrillary Tangles with Calcification" or "Kosaka-Shibayama Disease") OR AB ("Diffuse Neurofibrillary Tangles with Calcification" or "Kosaka- Shibayama Disease") OR TX ("Diffuse  Neurofibrillary Tangles with Calcification" or "Kosaka- Shibayama Disease") | **Search modes** - Find all my search terms | 8 |
| 24 | MH "Frontotemporal Lobar Degeneration+" | **Search modes** - Find all my search terms | 517 |
| 25 | TI ("Frontotemporal Lobar Degeneration" or FTLD) OR AB ("Frontotemporal Lobar Degeneration" or  FTLD) | **Search modes** - Find all my search  terms | 504 |
| 26 | MH "Frontotemporal Dementia+" | **Search modes** - Find all my search  terms | 458 |
| 27 | TI ("Frontotemporal Dementia" or DDPAC or "Dementia, Frontotemporal" or "Dementia, Frontotemporal, with Parkinsonism" or "Dementia,  Hereditary Dysphasic Disinhibition" or "Disinhibition- | **Search modes** - Find all my search terms | 1,633 |

|  | Dementia-Parkinsonism-Amyotrophy Complex" or "Disinhibition-Dementia-Parkinsonism-Amytrophy Complex" or "FTD-GRN" or "FTD-PGRN" or "FTDP- 17" or "FTLD with TDP-43 Pathology" or "FTLD-17 GRN" or "FTLD-TDP" or "Familial Pick's Disease" or "Frontotemporal Dementia with Parkinsonism" or "Frontotemporal Dementia with Parkinsonism-17" or "Frontotemporal Dementia, Ubiquitin-Positive" or "Frontotemporal Lobar Degeneration With Ubiquitin- Positive Inclusions" or "Frontotemporal Lobe Dementia" or "Frontotemporal Lobe Dementia (FLDEM) " or "GRN-Related Frontotemporal Dementia" or HDDD1 or HDDD2 or "Hereditary Dysphasic Disinhibition Dementia" or "Multiple System Tauopathy with Presenile Dementia" or "Semantic Dementia" or "Wilhelmsen-Lynch Disease" ) OR AB ( "Frontotemporal Dementia" or DDPAC or "Dementia, Frontotemporal" or "Dementia, Frontotemporal, with Parkinsonism" or "Dementia, Hereditary Dysphasic Disinhibition" or "Disinhibition- Dementia-Parkinsonism-Amyotrophy Complex" or "Disinhibition-Dementia-Parkinsonism-Amytrophy Complex" or "FTD-GRN" or "FTD-PGRN" or "FTDP- 17" or "FTLD with TDP-43 Pathology" or "FTLD-17 GRN" or "FTLD-TDP" or "Familial Pick's Disease" or "Frontotemporal Dementia with Parkinsonism" or "Frontotemporal Dementia with Parkinsonism-17" or "Frontotemporal Dementia, Ubiquitin-Positive" or "Frontotemporal Lobar Degeneration With Ubiquitin- Positive Inclusions" or "Frontotemporal Lobe Dementia" or "Frontotemporal Lobe Dementia (FLDEM) " or "GRN-Related Frontotemporal Dementia" or HDDD1 or HDDD2 or "Hereditary Dysphasic Disinhibition Dementia" or "Multiple System Tauopathy with Presenile Dementia" or "Semantic Dementia" or "Wilhelmsen-Lynch  Disease") |  |  |
| --- | --- | --- | --- |
| 28 | MH "Pick Disease of the Brain+" | **Search modes** -  Find all my search terms | 85 |
| 29 | TI ("Pick Disease of the Brain" or "Brain Atrophy, Circumscribed Lobar" or "Circumscribed Lobar Atrophy of the Brain" or "Dementia with Lobar Atrophy and Neuronal Cytoplasmic Inclusions" or "Lobar Atrophy (Brain)" or "Lobar Atrophy Of Brain" or "Lobar Atrophy of the Brain" or "Pick Disease" or "Pick Disease Of Brain" or "Pick's Disease" or "Picks Disease of Brain" ) OR AB ( "Pick Disease of the Brain" or "Brain Atrophy, Circumscribed Lobar" or "Circumscribed Lobar Atrophy of the Brain" or "Dementia with Lobar Atrophy and Neuronal Cytoplasmic Inclusions" or "Lobar Atrophy (Brain)" or "Lobar Atrophy Of Brain" or "Lobar Atrophy of the Brain" or "Pick Disease" or "Pick Disease Of Brain" or  "Pick's Disease" or "Picks Disease of Brain") | **Search modes** - Find all my search terms | 195 |

| 30 | MH "Lewy Body Disease+" | **Search modes** - Find all my search  terms | 754 |
| --- | --- | --- | --- |
| 31 | TI ( "Lewy Body Disease" OR "Cortical Lewy Body Disease" OR "Dementia, Lewy Body" OR "Diffuse Lewy Body Disease" OR "Lewy Body Dementia" OR "Lewy Body Disease, Cortical" OR "Lewy Body Disease, Diffuse" OR "Lewy Body Type Senile Dementia" ) OR AB ( "Lewy Body Disease" OR "Cortical Lewy Body Disease" OR "Dementia, Lewy Body" OR "Diffuse Lewy Body Disease" OR "Lewy Body Dementia" OR "Lewy Body Disease, Cortical" OR "Lewy Body Disease, Diffuse" OR "Lewy Body  Type Senile Dementia") | **Search modes** - Find all my search terms | 375 |
| 32 | S12 OR S13 OR S14 OR S15 OR S16 OR S17 OR S18 OR S19 OR S20 OR S21 OR S22 OR S23 OR S24 OR S25 OR S26 OR S27 OR S28 OR S29 OR  S30 OR S31 | **Search modes** - Find all my search terms | 73,624 |
| 33 | S7 AND S11 AND S32 | **Search modes** - Find all my search  terms | 320 |
| 34 | S7 AND S11 AND S32 | **Search modes** - Find all my search terms  **Limiters** - English language | 309 |
